# Supplementary material for: Prolonged cross-bridge binding triggers muscle dysfunction in a Drosophila model of myosin-based hypertrophic cardiomyopathy
Source: eLife. 2018 Aug 13;7:e38064. doi: 10.7554/eLife.38064 (PMC6141233; doi:10.7554/eLife.38064)
Supplement: Supplementary file 1. — All three fiber types were oscillated through ten 0.75% ML amplitude and 125 Hz contraction cycles. These parameters are the optimal power producing parameters for most of the control fibers (average results are shown in Table 2). Means ± S.E.M are reported. N = 12 for the control and homozygous fibers, 13 for heterozygous fibers. Student’s t-test with p<0.05 significantly different from control (*p<0.05, **p<0.01, ***p<0.001) or heterozygote (xp<0.05, xxp<0.01, xxxp<0.001). Full genotypes are shown in parentheses: PwMhc2 control (P{PwMhc2}/P{PwMhc2}; Mhc10/Mhc10); R146N-15/+ heterozygote (Mhc10/+; P{R146N-15}); R146N-15 homozygote (Mhc10/Mhc10; P{R146N-15}/P{R146N-15}). [file elife-38064-supp1.docx]

| Line | Power(W/m^3^) | Net Work (J/m^3^) | Work Gen. (J/m^3^) | Work Abs. (J/m^3^) |
| --- | --- | --- | --- | --- |
| *PwMhc2* control | 219 ±36 | 1.74 ±0.29 | 32.73 ±3.20 | 30.99 ±3.00 |
| *R146N-15*/+  heterozygote | 86 ±20** | 0.57 ±0.16** | 32.10 ±3.88 | 31.52 ±3.78 |
| *R146N-15*  homozygote | 20 ±15***^xx^ | 0.09 ±0.10***^x^ | 33.05 ±2.78 | 32.96 ±2.79 |
