# Supplementary material for: Prolonged cross-bridge binding triggers muscle dysfunction in a Drosophila model of myosin-based hypertrophic cardiomyopathy
Source: eLife. 2018 Aug 13;7:e38064. doi: 10.7554/eLife.38064 (PMC6141233; doi:10.7554/eLife.38064)
Supplement: Supplementary file 2. — Adult female flies were aged for two or seven days prior to flight testing. Transgenic flies were then assayed for the ability to fly up (U), horizontal (H), down (D) or not at all (N). Flight index = 6 U/T+ 4 H/T+ 2 D/T+ 0 N/T; T is the total number of flies tested, listed in parentheses. Flight index is mean ±S.E.M. Student’s t-test with p<0.05 significantly different from same age PwMhc2 (*p<0.05, **p<0.01, ***p<0.001) or from same fly line at 2 days of age (xp<0.05, xxp<0.01, xxxp<0.001). Full genotypes are shown in parentheses: PwMhc2 (P{PwMhc2}/P{PwMhc2}; Mhc10/Mhc10); R146N (Mhc10/Mhc10; P{R146N}/P{R146N}). [file elife-38064-supp2.docx]

| Line | Age (days) | Flight index 22°C |
| --- | --- | --- |
| *PwMhc2* | 2 | 4.6 ± 0.02 (148) |
| *R146N-11* | 2 | 1.3 ± 0.01 (121)*** |
| *R146N-15* | 2 | 1.5 ± 0.02 (120)*** |
| *R146N-28* | 2 | 1.3 ± 0.02 (118)*** |
| *PwMhc2* | 7 | 4.1 ± 0.01 (139) ^xxx^ |
| *R146N-11* | 7 | 0.84 ± 0.02 (131)*** ^xxx^ |
| *R146N-15* | 7 | 0.72 ± 0.01 (117)*** ^xxx^ |
| *R146N-28* | 7 | - 1. ± 0.02 (131)*** ^xxx^ |
