# Supplementary material for: Prolonged cross-bridge binding triggers muscle dysfunction in a Drosophila model of myosin-based hypertrophic cardiomyopathy
Source: eLife. 2018 Aug 13;7:e38064. doi: 10.7554/eLife.38064 (PMC6141233; doi:10.7554/eLife.38064)
Supplement: Supplementary file 3. — At least three hearts were analyzed per line and genotype, including three sections along the anterior-poster axis at least 10 microns apart per sample, and ≥3 images each for dorsal or ventral areas per section analyzed. Means ± S.E.M are reported. A one-way ANOVA with the Bonferroni correction determined that no statistically significant differences (p<0.05) exist for any of the comparisons within or between samples at the same or different ages. Full genotypes are shown in parentheses: PwMhc2/+ (P{PwMhc2}; Mhc1/+); R146N/+ (Mhc1/+; P{R146N}). [file elife-38064-supp3.docx]

| Line | Ventral  1 week | Dorsal  1 week | Ventral  3 weeks | Dorsal  3 weeks |
| --- | --- | --- | --- | --- |
| *PwMhc2*/+ | 2.50 ± 0.40 | 2.38 ± 0.28 | 2.92 ± 0.56 | 2.46 ± 0.25 |
| *R146N-15*/+ | 2.09 ± 0.21 | 2.09 ± 0.36 | 2.06 ± 0.27 | 2.16 ± 0.36 |
| *R146N-28*/+ | 1.62 ±0.15 | 1.46 ± 0.08 | 1.69 ± 0.32 | 1.75 ± 0.20 |
