# Supplementary material for: Prolonged cross-bridge binding triggers muscle dysfunction in a Drosophila model of myosin-based hypertrophic cardiomyopathy
Source: eLife. 2018 Aug 13;7:e38064. doi: 10.7554/eLife.38064 (PMC6141233; doi:10.7554/eLife.38064)
Supplement: Supplementary file 4. — Means ± S.E.M are reported. Two-way ANOVA results summarizing the statistical differences between the control and mutant flies can be found in Figure 7. No significant differences (p<0.05) in any cardiac physiological indices reported were noted between the two mutant lines via two-way ANOVA. Full genotypes are shown in parentheses: PwMhc2/+ (P{PwMhc2}; Mhc1/+); R146N/+ (Mhc1/+; P{R146N}). [file elife-38064-supp4.docx]

|  | **Heart Period** | **Systolic Interval** | **SI/HP** | **Diastolic Diameter** | **Systolic Diameter** | **Fractional Shortening** |
| --- | --- | --- | --- | --- | --- | --- |
| ***PwMhc2/+* 1 wk (N=42)** | 0.45 ± 0.02 | 0.19 ± 0.01 | 0.45 ± 0.02 | 65.68 ± 0.73 | 39.60 ± 0.46 | 0.40 ± 0.01 |
| ***R146N-15/+* 1 wk (N=41)** | 0.40 ± 0.02 | 0.21 ± 0.01 | 0.56 ± 0.02 | 52.13 ± 0.53 | 32.83 ± 0.45 | 0.37 ± 0.01 |
| ***R146N-28/+* 1 wk (N=40)** | 0.46 ± 0.02 | 0.23 ± 0.01 | 0.53 ± 0.02 | 51.96 ± 0.50 | 32.61 ± 0.47 | 0.37 ± 0.01 |
| ***PwMhc2/+*  3 wk (N=44)** | 0.65 ± 0.04 | 0.21 ± 0.01 | 0.35 ± 0.01 | 67.58 ± 0.80 | 43.49 ± 0.67 | 0.36 ± 0.01 |
| ***R146N-15/+* 3 wk (N=42)** | 0.66 ± 0.04 | 0.23 ± 0.01 | 0.40 ± 0.02 | 48.62 ± 0.64 | 34.76 ± 0.53 | 0.28 ± 0.01 |
| ***R146N-28/+* 3 wk (N=44)** | 0.68 ± 0.07 | 0.23 ± 0.01 | 0.43 ± 0.03 | 44.67 ± 0.54 | 31.34 ± 0.45 | 0.31 ± 0.01 |
